# Convergence of Plasma Metabolomics and Proteomics Analysis to Discover Signatures of High-Grade Serous Ovarian Cancer

**DOI:** 10.3390/cancers12113447

**Published:** 2020-11-19

**Authors:** Hee-Sung Ahn, Jeonghun Yeom, Jiyoung Yu, Young-Il Kwon, Jae-Hoon Kim, Kyunggon Kim

**Affiliations:** 1 Asan Institute for Life Sciences, Asan Medical Center, Seoul 05505, Korea; zaulim3@amc.seoul.kr (H.-S.A.); scarlet202@amc.seoul.kr (J.Y.); 2 Convergence Medicine Research Center, Asan Institute for Life Sciences, Asan Medical Center, Seoul 05505, Korea; nature8309@amc.seoul.kr; 3 The K-Clinic Royal HIFU Center, Seoul 06232, Korea; noyet@gmail.com; 4 Department of Obstetrics and Gynecology, Gangnam Severance Hospital, Yonsei University College of Medicine, Seoul 06237, Korea; 5 Department of Biomedical Sciences, University of Ulsan College of Medicine, Seoul 05505, Korea; 6 Clinical Proteomics Core Laboratory, Convergence Medicine Research Center, Asan Medical Center, Seoul 05505, Korea; 7 Bio-Medical Institute of Technology, Asan Medical Center, Seoul 05505, Korea

**Keywords:** liquid biopsy, ovarian cancer, metabolome, proteome, LC–MS/MS, FIA–MS/MS, biomarker, OMICS integrated analysis

## Abstract

**Simple Summary:**

In-time diagnosing ovarian cancer, intractable cancer that has no symptoms can increase the survival of women. The aim of this study was to discover biomarkers from liquid biopsy samples using multi-omics approach, metabolomics and proteomics for the diagnosis of ovarian cancer. To verify our biomarker candidates, we conducted comparative analysis with other previous published studies. Despite the limitations of non-invasive samples, our findings are able to discover emerging properties through the interplay between metabolites and proteins and mechanism-based biomarkers through integrated protein and metabolite analysis.

**Abstract:**

The 5-year survival rate in the early and late stages of ovarian cancer differs by 63%. In addition, a liquid biopsy is necessary because there are no symptoms in the early stage and tissue collection is difficult without using invasive methods. Therefore, there is a need for biomarkers to achieve this goal. In this study, we found blood-based metabolite or protein biomarker candidates for the diagnosis of ovarian cancer in the 20 clinical samples (10 ovarian cancer patients and 10 healthy control subjects). Plasma metabolites and proteins were measured and quantified using mass spectrometry in ovarian cancer patients and control groups. We identified the differential abundant biomolecules (34 metabolites and 197 proteins) and statistically integrated molecules of different dimensions to better understand ovarian cancer signal transduction and to identify novel biological mechanisms. In addition, the biomarker reliability was verified through comparison with existing research results. Integrated analysis of metabolome and proteome identified emerging properties difficult to grasp with the single omics approach, more reliably interpreted the cancer signaling pathway, and explored new drug targets. Especially, through this analysis, proteins (PPCS, PMP2, and TUBB) and metabolites (L-carnitine and PC-O (30:0)) related to the carnitine system involved in cancer plasticity were identified.

## 1. Introduction

Ovarian cancer is one of the fatal gynecological cancers, and the 5-year survival rate is approximately 47.7% which is the 8th lowest among cancers from 2008 to 2014 [1]. It is the fifth most common cause of cancer deaths in the United States, and about 14,000 women die from it each year [2]. Asymptomatic cancer disease progression occurs in the early stages of ovarian cancer with a 5-year survival rate of 92%, and symptoms appear in the later stages with a 5-year survival rate of 29% [3,4].

Liquid biopsy used to diagnose ovarian cancer is essential due to the difficulty of collecting ovarian tissue without using invasive means. Currently, screening with plasma cancer antigen 125 (CA-125) appears practical, but establishing the value of screening is challenging [3,4]. A novel biomarker for diagnosing the cancer is needed, and for this purpose, we applied two-omics, proteomic and metabolic, approaches to clinical plasma samples from the same person.

Tumor metabolism alterations were influenced by switching the activity of related enzymes or rearranging carcinogenic pathways induced by the genetic mutation or epigenetic changes [5,6,7]. Based on this assumption, recent comparative studies of ovarian cancer patients and healthy female volunteers have been conducted for the discovery of biomarkers in the tissue [8], plasma [9,10,11], and both [12]. Whereas, in recent years, there have been various studies related to ovarian cancer using proteomics, and among them were studies of serological markers discovery [13,14,15,16], studies of differences in biological mechanisms based on differential expression between normal and tumor tissues [17,18,19,20], and studies to integrate them with the genome [21,22,23,24,25]. Unfortunately, the combined study of metabolites and proteins in blood has not yet been investigated for ovarian cancer.

In this preliminary study, we carried out flow injection analysis (FIA) or LC–MS/MS profiling to discover blood-based metabolite or protein biomarker candidates for the diagnosis of ovarian cancer and we presented an analysis of the plasma metabolome and proteome in the 20 clinical samples (10 ovarian cancer and 10 female control subjects). In addition to independent protein and metabolite analysis, the emerging results were obtained through integrated analysis, and this information was useful for interpreting cancer signaling pathway activity and the exploration of new drug targets.

## 2. Results

### 2.1. Study Design

We collected plasma from 10 ovarian cancer (OC) patients and 10 female healthy controls (HC) and analyzed the proteome and metabolome of the samples. The demographic characteristics of the study attendants are summarized in Table 1. The mean (standard deviation) age of the subjects was 55.6 (12.5) years among the healthy female subjects and 59.7 (15.4) years in the ovarian cancer patients; this difference was not significant (*p* = 0.511). The patients with ovarian cancer had serous histological subtypes of stages 3 and 4.

### 2.2. Plasma ESI-LC–MS Based Metabolomic Analyses

**Figure 1:**
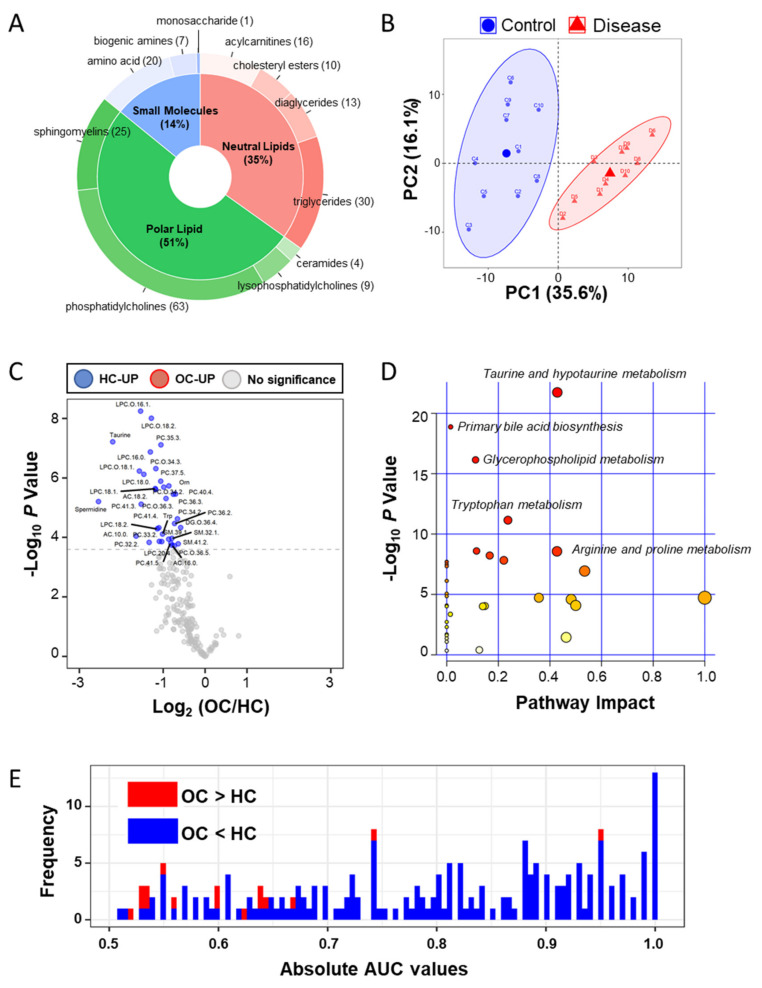
Targeted metabolic analysis by mass spectrometry in the clinical plasma samples. (**A**) Donut plot of 199 quantified metabolites in the AbsoluteIDQ p400 kits; (**B**) PCA analysis result of plasma metabolites in 20 clinical samples; (**C**) volcano plot of plasma metabolic data; volcano plots are depicted with the fold change of each metabolic amount and the *p* value was calculated by performing a Student’s *t*-test. Blue circles are less than a Bonferroni-corrected *p*-value < 0.05, indicating a significantly increased 34 metabolites in HC. Gray circles are plasma metabolites without statistical meaning. (**D**) Pathway enrichment analysis in the MetaboAnalyst 4.0 tool; the y-axis represents the *p* value, the x-axis represents the impact value; the top five pathways are represented as text based on the *p*-value. The color and size of each circle represents *p*-values and pathway impact values, respectively. (**E**) Histogram of 199 absolute area under curve values in univariate ROC analysis. Red bar indicates the high mean value in ovarian cancer and the blue bar indicates the low mean value.

In metabolites, we used the AbsoluteIDQ^®^ p400 HR Kit (Biocrates Life Science AG, Innsbruck, Austria) for absolutely quantifying 408 metabolites in the plasma samples. Quality control was performed according to the Biocrates manufacturer’s sample measurement method (Innsbruck, Austria), and metabolite quantitation data were filtered based on the limit of determination (LOD) and the limit of quantification. Then, we absolutely quantified 199 metabolites, 20 amino acids, 7 biogenic amines, 1 monosaccharide, 16 acylcarnitines (AC), 13 diglycerides (DC), 30 triglycerides, 9 lysophosphatidylcholines (LPS), 63 phosphatidylcholines (PC), 25 sphingomyelins (SM), 4 ceramides, and 10 cholesteryl esters (Figure 1A and Table S1). Two groups were clearly segregated by principle components 1 (35.6%) and 2 (16.1%; Figure 1B). To find the differentially abundant plasma metabolites (DAMs), fold-changes and Bonferroni-corrected *p*-values (*q*-value) were calculated by Student’s *t*-test analysis of the two groups. A volcano plot showing log2-fold-changes against minus log10 *p*-values identified 34 metabolites as being upregulated in the HC (*q*-value < 0.05; Figure 1C and Table S1). The pathway enrichment analysis based on metabolite quantitative alterations was performed by the MetaboAnalyst 4.0 (http://www.metaboanalyst.ca) [26] (Figure 1D). The HC-upregulated proteins were highly involved in “Taurine and hypotaurine metabolism”, “Primary bile acid biosynthesis”, “Glycerophospholipid metabolism”, and “Tryptophan metabolism”. In addition, to confirm the diagnostic ability, we performed univariate receiver operating characteristic (ROC) analysis of metabolites against the occurrence of ovarian cancer (Figure 1E and Table S2). In the results, 116 metabolites were significant (*p* < 0.05), and 36 metabolites represented more than 0.95 of the area under the curve (AUC) value.

### 2.3. Plasma ESI-LC–MS/MS Proteomic Analyses

**Figure 2:**
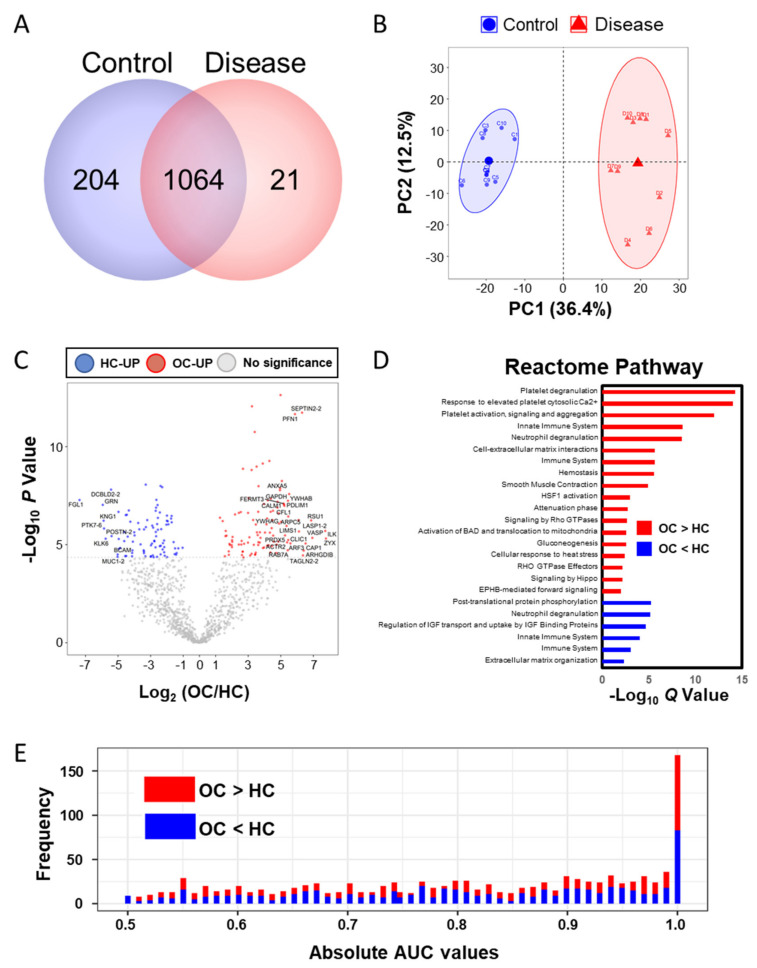
Proteomic analysis by LC–MS/MS. (**A**) The number of identified proteins in the two groups (control and disease); (**B**) PCA analysis result of the plasma proteins; (**C**) volcano plot of the plasma proteomic data; red circles are less than the Bonferroni corrected *p*-value < 0.05, indicating a significantly increased 108 proteins in OC. Blue circles are less than a Bonferroni-corrected *p*-value < 0.05, indicating a significantly increased 89 proteins in HC. (**D**) Reactome pathway enrichment analysis; red bar indicates that the pathways were enriched in the upregulated proteins in OC, and blue indicates that the pathways are enriched in the upregulated proteins in HC. The size of the bar is the Q value. (**E**) Histogram of 1124 absolute area under curve values in univariate ROC analysis.

A total of 1289 proteins were identified in a total of 20 LC–MS/MS measurements (Figure 2A). By label free quantification (LFQ), we eliminated proteins bound to the MARS14 affinity column and measured less than three times in one group, then we selected filtered 1124 proteins that were normalized by width adjustment and missing value imputation and we performed principle component analysis (PCA) analysis (Figure 2B and Table S3). Similarly, we applied the above-mentioned metabolite statistical analysis to 1124 proteins to discover the differential abundant plasma proteins (DAPs) and we identified 108 proteins as being upregulated in the OC and 89 proteins in the HC (Bonferroni-corrected *p* < 0.05; Figure 2C and Table S4). By using g:Profiler [27], the Reactome pathways, which differed significantly between the two groups, are shown in Figure 2D. The OC-upregulated plasma proteins were enriched in the eight functional categories. First, platelets are known to increase ovarian cancer growth or activate metastasis [28,29,30,31,32], and functional terms related to this include “Platelet degranulation”, “Response to elevated platelet cytosolic Ca2^+^”, and “Platelet activation, signaling, and aggregation”. Second, the immune response in the tumor environment around ovarian cancer is related to the patient’s prognosis [33,34], and related terms include “Immune System”, “Innate Immune System”, “Neutrophil degranulation”, and “Attenuation phase. Third, it is related to the mechanisms related to the energy metabolism of ovarian cancer [35,36,37], and the related term is “Gluconeogenesis”. Fourth, “Hemostasis” has been linked to ovarian cancer [38,39]. Fifth, it is known that ovarian smooth muscle tumors and ECM-related proteins regulate the cancer environment, and related terms are “Cell–extracellular matrix interactions” and “Smooth Muscle Contraction”. Sixth, ovarian cancer that responds to external stimuli or stress causes “HSF1 activation” and “cellular response to heat stress” [40,41,42,43]. Seventh, there are terms “Activation of BAD and translocation to mitochondria” and “Signaling by Hippo” as the mechanisms involved in cancer and the surrounding normal cells for signaling for ovarian cancer survival [44,45,46,47]. Finally, the terms “Signaling by Rho GTPases”, “EPHB-mediated forward signaling”, and “RHO GTPase Effectors” related to cross-talk between Ras and Rho signaling, well known as ovarian cancer signaling, were enriched [48,49,50]. The HC-upregulated proteins were highly involved in “Post-translational protein phosphorylation”, “Neutrophil degranulation”, “Regulation of Insulin-like Growth Factor (IGF) transport and uptake by IGF Binding Proteins”, “Innate Immune System”, “Immune System”, and “Extracellular matrix organization”. Most of these terms were also elevated in ovarian cancer patients except for the IGF-related function, which was related to the risk of ovarian cancer [51,52]. Then, we performed univariate ROC analysis of proteins against the occurrence of ovarian cancer (Figure 2E and Table S4). We found 702 proteins were significant (*p* < 0.05), and 161 proteins represented more than 0.95 of the AUC value.

### 2.4. Ingenuity Pathway Analysis (IPA) of the Integrated Metabolites and Proteins

**Figure 3:**
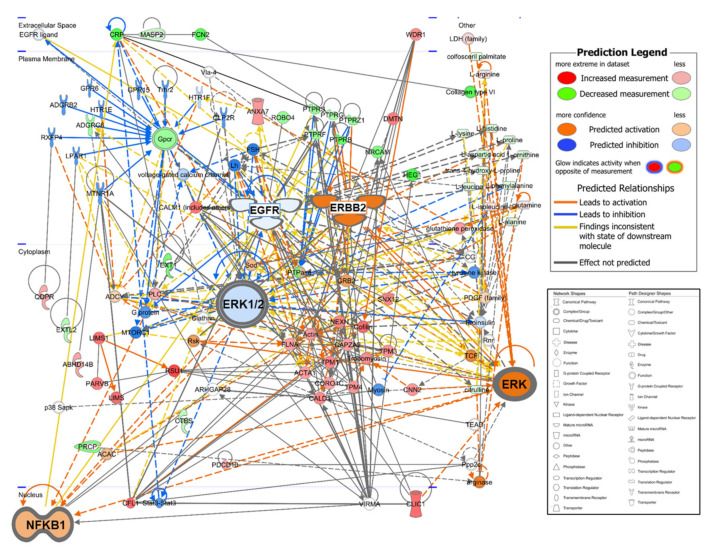
Merging network associated with the EGFR/ERBB2 signaling pathway by IPA-identified three networks (#1, #5, and #12 in Table S6). EGFR and ERK1/2 are weakly downregulated, and NFKB1 and ERBB2 are strongly upregulated in ovarian cancer patients. The network shapes show the categories of the proteins. In the IPA network, red indicates ovarian cancer upregulation, green, downregulation, orange, predicted upregulation, and blue, predicted downregulation and the color intensity indicates the magnitude of the relative change in protein expression.

The 199 quantified metabolites and 197 DAPs were used for integration analysis in IPA. As a result of canonical pathway analysis, the top five significantly different pathways were “tRNA Charging”, “Remodeling of Epithelial Adherens Junctions”, “Integrin Signaling”, “RhoA Signaling”, and “Superpathway of Citrulline Metabolism” (Table S5). In the network function analysis, IPA identified 15 highly enriched pathway networks (Table S6). Then, we applied the Molecular activity predictor in IPA to predict the consequences of these pathway changes for biological function. Three networks (Network #1, #5, and #12) were linked to EGFR/ERBB2 signaling pathways that are well-known to have a major role in ovarian cancer [53,54,55] (Figure 3). It can be inferred that the EGFR signal is inactivated, and the ERBB2 (alternative gene name: HER2) signal is activated while NFkB is activated to promote tumor-cell proliferation and survival [56,57,58]. Network #2 represents a general cancer signaling pathway from hypoxia-inducible factor 1 (HIF-1) under tumor hypoxia, and it is known that targeting this network could overcome anticancer drug resistance [59,60]. Network #3 represented reduced TGFBI abundance to derive the PI3 K/AKT pathway inhibition, a common manifestation in cancer [61], and the JAK/STAT signaling pathway, which is proposed as a new target for ovarian cancer anticancer drug resistance [62,63], is dysregulated in network #8 and other signaling pathways associated with ovarian are shown in Figure S1.

### 2.5. Integration Analysis for Discovering Clinical Markers

**Figure 4:**
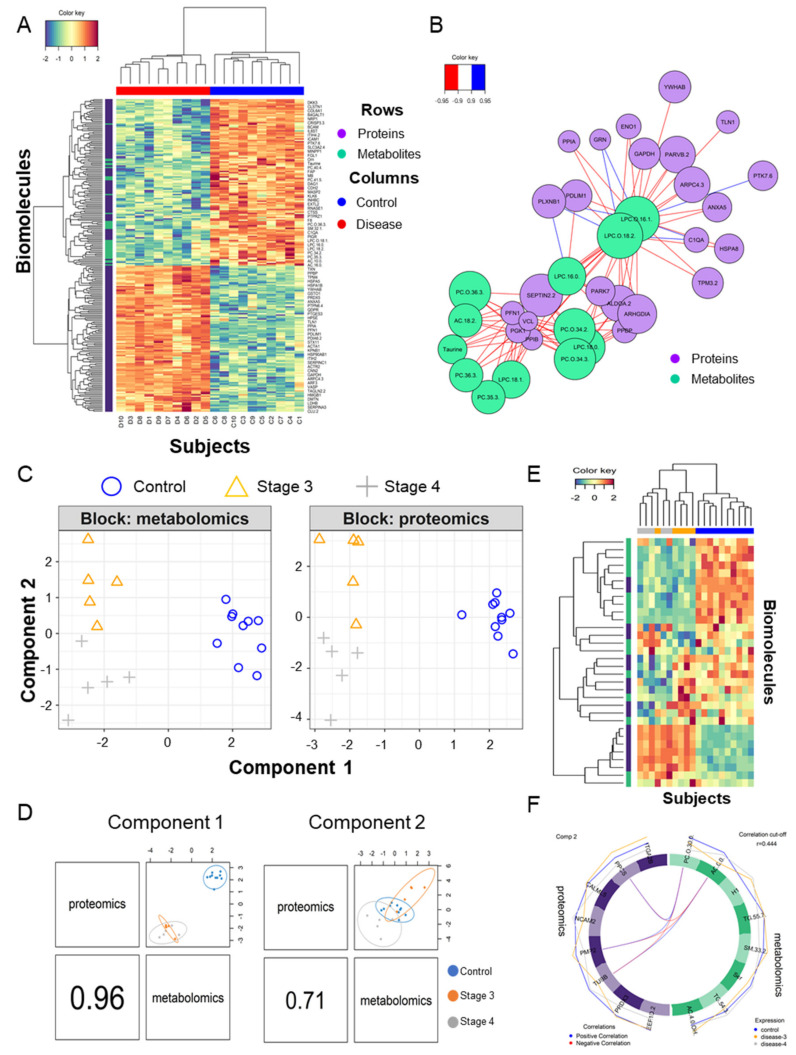
Multiomics combined analysis of metabolome and proteome. (**A**) Heatmap of hieratical clustering of 34 DAMs and 197 DAPs in the two groups (control and disease); (**B**) Diagram of the correlation between DAMs and DAPs with a correlation between them with an absolute value of 0.9 or higher. (**C**) Blocked sparse PLA-DS analysis of metabolites and proteins that divide the control, stage 3, and stage 4 groups; (**D**) Scatter plot of the 1st component of the proteome and metabolome in the three groups; the second component also appears the same. (**E**) Heatmap of hieratical clustering of eight metabolites and proteins contributing to components 1 and 2, respectively; (**F**) Diagram of the correlation between eight metabolites and eight proteins contributing to component 2 with a correlation between them with an absolute value of 0.444 or higher.

Assuming a null correlation between the proteome and metabolome, we incorporated two biomolecular domains by using sparse multiblock partial least square discriminant analysis in Data Integration Analysis and Biomarker discovery using Latent cOmponents (DIABLO) [64]. A heatmap of two-dimensional biomolecules is displayed in Figure 4A. A correlation plot between the abundances of the metabolites and proteins is shown in Figure 4B. Polar lipids, LPC and PC were highly correlated with 24 proteins (|*r*| > 0.9), which function in cadherin binding, were located in focal adhesion, cell-substrate junction, and extracellular vesicles by analyzing g:Profiler [27] (Figure 4B). To overcome the small sample size bias, we compared the DAMs in several other metabolic biomarker studies (Table 2). In the other two blood-derived ovarian cancer studies [9,11], similar to the results of this study, the abundances of two types of polar lipids, LPC and PC, were generally significantly lower in ovarian cancer patients. In particular, the amount of LPC (18:0) showed the same pattern in all three studies. Otherwise, in metabolic analysis studies in tissues [8], the amount of polar lipids and taurine were significantly high as opposed to in the blood samples.

In proteins, unlike metabolites, some of the proteins overexpressed in ovarian cancer tissues could be released into the plasma [65], and in this respect, we examined whether DAPs are differentially expressed in ovarian cancer tissues. In a recent study [23], they integrated the proteogenomic signature from the high-grade serous ovarian cancer tissues. Our 137 proteins out of 197 DAPs were subjected to Kaplan–Meier (KM) analysis in terms of overall survival (OS; *n* = 169), disease-free survival (DFS; *n* = 145), and platinum-free interval (PFI; *n* = 126). Independently, 61 proteins in 5-year OS analysis, 74 proteins in 5-year DFS survival analysis, and 56 proteins in PFI were significant. Among them, 24 proteins, namely ACTR2, ANPEP, ANXA5, ARF3, ARHGDIA, ARPC4, COPB1, CRP, ENO1, DPS, GSTO1, ILK, LASP1, LDHA, MSLN, NEXN, PDIA4, PDLIM5, PTPRG, SERPINC1, TAGLN, TAGLN2, TPM3, and TPM4 were commonly statistically significant in the three survival analyses. Unfortunately, ovarian cancer blood protein markers mainly consist of antibody-based methods with a focus on CA-125 and there are fewer than five markers, so there was no comparable data with more than a hundred proteins [66,67,68,69,70].

**Figure 5:**
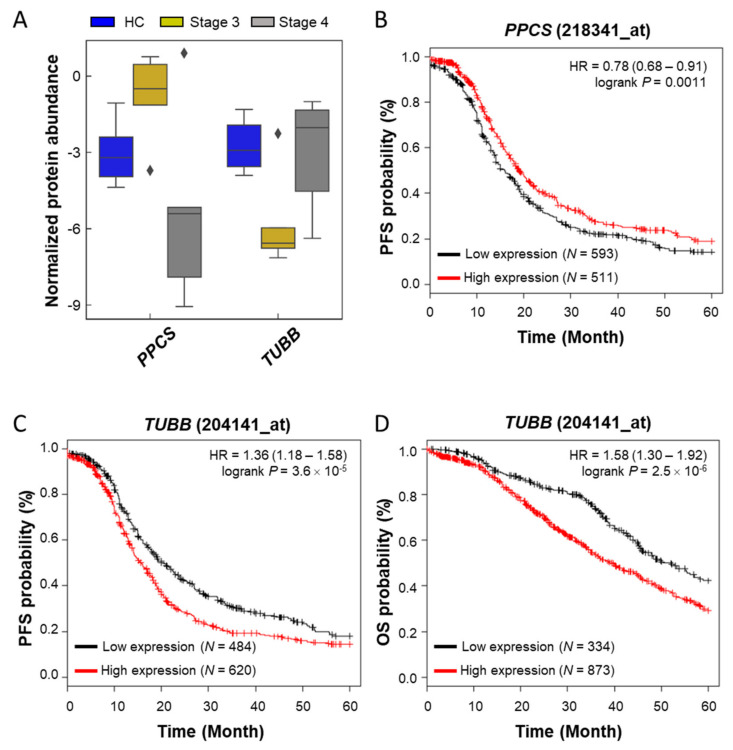
Boxplot and survival curves of two genes (*PPCS* and *TUBB*) selected as prognostic markers candidates. (**A**) A boxplot for the protein normalized abundances of two proteins in three groups obtained in this study; (**B**) Kaplan–Meier plot of *PPCS* (Probe set image ID: _at) in progression-free survival (PFS); (**C**) survival curve of *TUBB* (Probe set image ID: _at) in PFS; (**D**) survival curve of *TUBB* in overall survival (OS).

Patients with stage 4 ovarian cancer have metastases to other organs. To find the features from biomolecules, we divided the ovarian cancer into different stages and performed DIABLO analysis in three groups (control, stage 3, and stage 4) and built a horizontal integration partial least squares-discriminant analysis model (Figure 4C). Its first component classified the control and disease and its second component classified stage 3 and stage 4 regardless of the control (Figure 4D). Two components contained the eight metabolites and proteins selected for each on the heatmap (Figure 4E). Correlation analysis was performed by focusing on proteins and metabolites with a second component that divided stages 3 and 4, which differentiated cancer metastasis, and this is shown as a circus plot (Figure 4F). Three proteins, phosphopantothenate-cysteine ligase (PPCS), myelin P2 protein (PMP2), and tubulin beta chain (TUBB), were significantly correlated with L-carnitine (AC.0.0) and PC-O (30:0). The carnitine system is related to cancer metabolic plasticity [71] and acetylation of carnitine facilitates myelination of regenerated axons after peripheral nerve injuries with physical binding to PMP2 and TUBB [72]. In order to validate prognostic protein marker candidates, survival analysis was performed on the webpage Kaplan–Meier plotter (KM plotter, http://kmplot.com/) with mRNA expression and a progression-free survival (PFS) period [73] for 1232 serous ovarian cancer patients. The proteins were not mapped to the above ovarian tissue protein survival analysis. Anyway, based on the 5-year PFS, *CALML5*, *ITGA2 B*, *PMP2*, *PPCS,* and *TUBB* out of eight genes in the second component were statistically significant in two subtypes as low or high risk groups (hazard ratio (HR), 1.31, 1.16, 1.29, 0.78, and 1.36; log-rank test: *p* < 0.05). Among them, only two genes, *PPCS* and *TUBB*, matched the risk in the same direction in the amount of plasma and mRNA as the disease progressed (Figure 5A–C). In addition, *TUBB* also showed statistical significance against 5-year overall survival (HR, 1.58; log-rank test: *p* < 0.05; Figure 5D).

## 3. Discussion

In the current study, metabolite biomarker candidates are generally reduced in the plasmas of ovarian cancer patients. Plasma deprivation of them is indirect evidence that ovarian cancer cells consumed these metabolites supplied from the blood. The process of absorbing the metabolites necessary for the growth of cancer cells supplied from the culture supplement was demonstrated in vitro [74,75,76]. Releasing taurine occurred in ovarian cancer cells sensitive to cisplatin [77]. Tryptophan is used for direct catalytic reactions of oncogenic enzyme, which is a tryptophan-degrading enzyme indoleamine 2,3-dioxygenase, immunoescape mechanism of tumor cells [78,79]. Likewise, ornithine decarboxylase launches the polyamine biosynthetic pathway by using ornithine, and is activated with high expression in cancer cells to increase the susceptibility of tumor development to changes in polyamine levels and by transforming the response to cytokines, abnormal oncogenic gene expression, and tumor promoter mutations [80,81]. Spermidine, a polyamine compound, could suppress the cancer cells by inducing autophagic apoptosis activation [82,83] but is found at lower levels in ovarian cancer patients’ plasmas. The alteration of lipids including LPC, PC, and SM cause ovarian carcinogenesis, and the risk of ovarian cancer with plasma lipids has been identified [84]. LPC is a bioactive proinflammatory lipid produced by pathological activities [85] and rewired storage and metabolism in ovarian cancer cells after treating with anti-VEGF agents [86]. PC is a major component of biological membranes and plays a role in cell proliferation and survival [87]. It has been reported that the alteration of PC metabolism in cancer cells is a signature of tumor progression and could be a target for anticancer agents [88]. In particular, the aberrant reaction is triggered by phospholipase C activation in epithelial ovarian cancer cells [89,90]. SM consists of a phosphocholine head group and ceramide contained sphingosine and fatty acids and its subcellular location is correlated with cholesterol [91]. It is important to acquire a resistance to anticancer agents since ovarian cancer cells provoke an aberrant SM mechanism that involves the promotion of the catabolism of ceramide, and the production and accumulation of ceramide [92,93].

In the study of biomarker discovery in blood, many researchers have tried to explain the causal relationship with disease by identifying their biological function using differential abundant molecules. Interestingly, in this study, it was found that the interpretation of the cancer signaling pathways contrasts with the network analysis using only the protein and the analysis involving metabolites. Between the two analysis results, the activation of NFkB, ERK1/2, estrogen receptor, and HIF-1 signaling pathways had the opposite results, except for the ERK and AKT signaling pathway. This result may reveal a blind spot in the analysis of widely used blood protein-based disease-associated pathways. On the other hand, by integrating metabolites and proteins, this multiomics analysis aimed to find emerging properties that were difficult to see in a one omics approach. Through the comparative analysis function of IPA, we discovered key emerging pathway upstream regulators that were indicators for discovering new drug targets, and toxicological analysis showed that the patient’s liver, heart, and renal function was damaged. One of the emerging canonical pathways is “Glutathione-mediated Detoxification” (activation z-score: -0.447) in which glutathione is involved in the regulation of ROS in cancer progression [94]. In the new drug target discovery, it was found that ERBB2 is activated in patients with ovarian cancer, and for BMS-, pirotinib, allitinib, poziotinib, erlotinib, sapitinib, osimertinib, lapatinib, nordihydroguaiaretic acid, and afatinib (activation score > 0.7), which can inhibit ERBB2, were found among upstream regulators found only in the integrated analysis.

This retrospective study focused on the metabolic and proteomic analysis of OC patients, and it has several limitations. The patient population was homogenous, enrolled at a single-center and of a small sample size. Plasma markers in patients with stage 3–4 might have divergent trends according to the character of peri- or postoperative adjuvant therapy. Therefore, these results require further validation in multicenter cohorts including larger numbers of patients to evaluate their applicability to broader populations with ovarian cancer.

## 4. Materials and Methods

### 4.1. Sample Subjects

All plasma specimens in this study were obtained with appropriate consent and approval of the institutional review board of Yonsei University Gangnam Severance Hospital (IRB number: 3–2018-0166, approved on 18 July 2018). All data were collected anonymously. This study was exempt from obtaining informed consent by the IRB committee. Plasma samples were obtained preoperatively from 10 OC patients and 10 female HC subjects and provided by the Korea Gynecologic Cancer Bank through Bio and Medical Technology Development Program of the Ministry of the National Research Foundation (NRF) funded by the Korean government (MSIT) (NRF-2017 M3 A9 B). The clinical data of the 20 participants are summarized in Table 1. All samples were frozen in liquid nitrogen and were stored at −80 °C until analysis.

### 4.2. Metabolite Analysis

The targeted metabolomics evaluation used electrospray ionization liquid chromatography–tandem mass spectrometry (ESI-LC–MS/MS) and flow injection analysis–tandem mass spectrometry (FIA–MS/MS) techniques with the AbsoluteIDQ™ p400 kit (BIOCRATES Life Sciences AG, Innsbruck, Austria) to analyze 10 µL of plasma from each patient. The assay allows for simultaneous quantification of 408 metabolites out of 10 µL plasma, including 21 amino acids (19 proteinogenic amino acids, citrulline and ornithine), 21 biogenic amines, hexose (sum of hexoses—about 90–95% glucose), 55 acylcarnitines, 18 diglycerides, 42 triglycerides, 24 lysophosphatidylcholines and 172 phosphatidylcholines, 31 sphingolipids, 9 ceramides, and 14 cholesteryl esters (Table S1). Plasma samples (10 μL), blanks, calibration standards, and quality controls were prepared according to the manufacturer’s manual instructions. All amino acids and biogenic amines were derivatized with phenylisothiocyanate and quantified by multiple reaction monitoring (MRM) techniques with internal standards. MRM–MS analyses were carried out on an API 4000 LC–MS/MS System (AB Sciex Deutschland GmbH, Darmstadt, Germany) equipped with a 1200 Series HPLC (Agilent Technologies Deutschland GmbH, Boeblingen, Germany) controlled by the Analyst 1.5.1 software. Otherwise, the remaining 366 metabolites were measured by FIA–MS/MS which were analyzed on a Thermo Scientific UltiMate 3000 Rapid Separation Quaternary HPLC System (Thermo Scientific, Madison, WI, USA), connected to a QExactive™ Plus Hybrid Quadrupole-Orbitrap™ Mass Spectrometer (Thermo Scientific, Waltham, MA, USA). Following this, the concentrations of the metabolites were generated by Biocrates MetIDQ software. The quality control of the experiment was also validated using the Biocrates software (version 5, MetIDQ, Biocrates, Innsbruck, Austria).

### 4.3. Proteomic Sample Preparation

Plasma samples were sequentially prepared by high abundant plasma protein depletion and trypsin/Lys-C digestion steps. At first, we depleted the high abundant plasma proteins by a Multiple Affinity Removal Column Human 14 (100 × 4.6 mm; MARS14, Agilent, CA, USA) column equipped in the HPLC systems. We digested the proteins to peptides by the amicon-adapted enhanced FASP method [95] and salts were removed sequentially by the C18 desalting cartridge (Sep-Pak C18 1 cc, Waters, USA). First, 40 μL of plasma was injected into the MARS14 depletion column in which the top 14 abundant proteins (albumin, IgA, IgG, IgM, a1-antitrypsin, a1-acid glycoprotein, apolipoprotein A1, apolipoprotein A2, complement C3, transferrin, a2-marcoglobulin, transthyretin, haptoglobin, and fibrinogen) were depleted. For this, the mixture was 4-fold diluted with a proprietary “Buffer A” and loaded onto a MARS14 column on a Shimadzu HPLC system. The unbound fraction was buffer-exchanged into 8 M urea in 50 mM Tris (pH 8) and 20 mM dithiothreitol and concentrated through ultrafiltration using a Vivaspin 500 3 kDa cutoff filter (Sartorius, Goettingen, Germany) to approximately 50 μL and then transferred to a new filter unit (Nanosep, 30 kDa; Pall Corporation, NY, USA). We added 200 μL of 8 M urea in 50 mM Tris (pH 8.5) and centrifuged it at 14,000× *g* for 15 min repeated twice. We discarded the flow-through from the collection tube. We then added 100 µL of iodoacetamide solution and mixed it at 600 rpm in a thermo-mixer for 1 min and incubated it without mixing for 20 min. We centrifuged the filter units at 14,000× *g* for 10 min. Then, we added 100 µL of 8 M urea in 100 mM ammonium bicarbonate (ABC) to the filter unit and centrifuged it at 14,000× *g* for 15 min. We repeated this step twice. Then, we added 100 µL of ABC to the filter unit and centrifuged it at 14,000× *g* for 10 min. We repeated this step twice. We added 40 µL ABC with Lys-C/trypsin (enzyme to protein ratio 1:25) and mixed it at 600 rpm in a thermo-mixer for 1 min. We incubated the units in a wet chamber at 37 °C for 12 h. We transferred the filter units to new collection tubes and centrifuged the filter units at 14,000× *g* for 10 min. Then, we added 40 µL of 0.5 M NaCl and centrifuged the filter units at 14,000× *g* for 10 min. Formic acid was then added to a final concentration of 0.3% to stop the digestion reaction. The peptide mixture was then desalted with a Sep Pak C-18 cartridge (Waters, Milford, MA, USA), lyophilized with a cold trap (CentriVap Cold Traps, Labconco, Kansas City, MO, USA) and stored at −80 °C until use.

### 4.4. Nano-LC-ESI–MS/MS Proteomic Analysis

Digested peptides were separated using a Dionex UltiMate 3000 RSLCnano system (Thermo Fisher Scientific). Tryptic peptides from the bead column were reconstituted in 0.1% formic acid and separated on an Acclaim™ Pepmap 100 C18 column (500 mm × 75 μm i.d., 3 μm, 100 Å) equipped with a C18 Pepmap trap column (20 mm × 100 μm i.d., 5 μm, 100 Å; Thermo Scientific, USA) over 200 min (350 nL/min) using a 0–48% acetonitrile gradient in 0.1% formic acid and 5% DMSO for 150 min at 50 °C. The LC was coupled to a Q Exactive™ Plus Hybrid Quadrupole-Orbitrap™ mass spectrometer with a nano-ESI source. Mass spectra were acquired in a data-dependent mode with an automatic switch between a full scan and 20 data-dependent MS/MS scans. The target value for the full scan MS spectra, selected from 350 to 1800 mass to charge ratio (m/z), was 3,000,000 with a maximum injection time of 100 ms and a resolution of 70,000 at m/z 400. The selected ions were fragmented by higher-energy collisional dissociation in the following parameters: 2 Da precursor ion isolation window and 27% normalized collision energy. The ion target value for MS/MS was set to 1,000,000 with a maximum injection time of 50 ms and a resolution of 17,500 at m/z 400. Repeated peptides were dynamically excluded for 20 s. All MS data have been deposited in the PRIDE archive (www.ebi.ac.uk/pride/archive/) [96] under Project PXD.

### 4.5. Protein Database Searching and Label Free Quantitation

The acquired MS/MS spectra were searched using the SequestHT on Proteome discoverer (version 2.2, Thermo Fisher Scientific, USA) against the SwissProt human database (1 May 2017). The search parameters were set as default including cysteine carbamidomethylation as a fixed modification, and n-terminal acetylation and methionine oxidation as variable modifications with two miscleavages. Peptides were identified based on a search with an initial mass deviation of the precursor ion of up to 10 ppm, with the allowed fragment mass deviation set to 20 ppm. When assigning proteins to peptides, both unique and razor peptides were used. Label-free quantitation (LFQ) was performed using peak intensity for unique peptides of each protein [97].

### 4.6. Statistical Metabolomic and Proteomic Analyses

In the metabolomic data, 199 out of 408 metabolites were excluded from further analysis as they exceeded the quality control (with no at zero concentration and concentrations below LOD in 10% and below of all samples). In the proteome data, LFQ was performed with the missing data filling of gaussian imputation separately for each column at parameter settings width = 0.3 and down-shift = 1.8 and normalization by width adjustment in the perseus software [98]. Data were analyzed using RStudio (version 1.1.456) including R (version 3.6.0). Statistical R software packages included ggplot2 for drawing box and volcano plots, stats for calculating *t*-test, pROC for ROC analysis, mixOmics for the integration of metabolic and proteomic data, drawing the heatmap, correlation and circus plots, and pcamethods for PCA analysis.

### 4.7. Pathway Analysis

In the metabolomic data, DAMs in the OC and HC groups were analyzed using Pathway analysis in the Metanalyst 4.0. We selected the parameters Over Representation Analysis: “Hypergeometric Test”, Pathway Topology Analysis: “Relative-betweeness Centrality”, Mammals: “Homo sapiens (KEGG)”, and “Use all compounds in the selected pathways”. In proteomic data, molecular reactions of DAPs in the OC and HC groups were annotated by g:Profiler. It was performed twice with a protein list with significantly higher amounts for each group. The input data were the UniProtKB accession codes of the proteins. We set the two parameters significance threshold: g:SCS, threshold: 0.05. Data sources were selected by Reactome in the biological pathways. In the integration analysis, ingenuity pathway analysis (Ingenuity System Inc, Redwood City, CA, USA) was used to carry out a core analysis of the integrated 199 quantified metabolites and DAPs. In the uploaded data, the PubChem IDs for metabolite and the UniProtKB IDs for the protein were entered, and both contained log2 fold-changes between OC and HC. Based on the network results, an integrated analysis was performed using a molecular activity predictor tool.

## 5. Conclusions

In this study, we integrated plasma metabolome and proteome data for discovering signatures of high-grade serous ovarian cancer. Ovarian cancer-related signaling pathways were more confidently interpreted by a multiomics approach beyond the limitations of single-omics analysis, and new drug candidates were found.

## Figures and Tables

**Table 1:** Demographic and clinical variables of the clinical samples.

Variable	Healthy Controls (*n* = 10)	Ovarian Cancer Patients (*n* = 10)	*p*-Value ^1^
Age (years)	55.5 ± 12.5	59.7 ± 15.4	0.511
Histology (*n*)	NA	10	-
Serous	NA	10	-
FIGO ^2^	-	-	-
1 a	-	0	-
1 b	-	0	-
1 c	-	0	-
2 a	-	0	-
2 b	-	0	-
2 c	-	0	-
3 a	-	2	-
3 b	-	0	-
3 c	-	3	-
4	-	5	-

^1^ Calculated by Student’s independent *t*-test. ^2^ Federation of International of Gynecologists and Obstetricians.

**Table 2:** Meta-analysis of DAMs with already published studies.

Name	Current Study		Plasma [11]		Plasma [9]		Tissue [8]	
	FC (OC/HC)	*p*	FC (OC/HC)	*p*	FC (OC/HC)	*p*	FC (OC/HC)	*p*
Ornithine	0.55	1.85 × 10^−6^	-	-	-	-	4.00	1.00 × 10^−4^
Tryptophan	0.50	7.68 × 10^−5^	−	-	-	-	2.42	1.00 × 10^−4^
Spermidine	0.17	6.26 × 10^−6^	-	-	-	-	2.25	3.00 × 10^−4^
Taurine	0.22	6.14 × 10^−8^	-	-	-	-	3.00	4.00 × 10^−4^
AC(10:0)	0.32	9.09 × 10^−5^	-	-	-	-	-	-
AC(16:0)	0.58	1.82 × 10^−4^	-	-	-	-	-	-
AC(18:2)	0.44	2.47 × 10^−6^	-	-	-	-	-	-
DG-O(36:4)	0.67	4.65 × 10^−5^	-	-	-	-	-	-
LPC(16:0)	0.40	1.34 × 10^−7^	-	-	-	-	1.00	2.23 × 10^−2^
LPC(18:0)	0.36	7.58 × 10^−7^	0.74	8.77 × 10^−3^	0.74	2.00 × 10^−4^	1.50	2.43 × 10^−2^
LPC(18:1)	0.44	2.29 × 10^−6^	-	-	0.72	2.90 × 10^−5^	1.50	2.43 × 10^−2^
LPC(18:2)	0.45	5.15 × 10^−5^	-	-	-	-	2.00	2.65 × 10^−2^
LPC(20:4)	0.49	1.38 × 10^−4^	-	-	-	-	1.00	2.88 × 10^−2^
LPC-O(16:1)	0.34	5.65 × 10^−9^	-	-	-	-	-	-
LPC-O(18:1)	0.34	5.85 × 10^−7^	-	-	-	-	-	-
LPC-O(18:2)	0.41	9.80 × 10^−9^	-	-	-	-	-	-
PC(32:2)	0.40	1.47 × 10^−4^	0.59	7.70 × 10^−5^	-	-	-	-
PC(33:2)	0.47	1.35 × 10^−4^	-	-	-	-	-	-
PC(34:2)	0.63	2.37 × 10^−5^	-	-	-	-	-	-
PC(35:3)	0.48	7.74 × 10^−8^	-	-	-	-	-	-
PC(36:2)	0.60	3.41 × 10^−5^	0.81	1.29 × 10^−2^	-	-	-	-
PC(36:3)	0.59	3.55 × 10^−6^	-	-	0.87	1.47 × 10^−2^	-	-
PC(37:5)	0.48	1.30 × 10^−6^	-	-	-	-	-	-
PC(40:4)	0.61	3.49 × 10^−6^	-	-	-	-	-	-
PC(41:3)	0.35	7.60 × 10^−6^	-	-	-	-	-	-
PC(41:4)	0.47	4.75 × 10^−5^	-	-	-	-	-	-
PC(41:5)	0.57	1.56 × 10^−4^	-	-	-	-	-	-
PC-O(34:2)	0.51	2.05 × 10^−6^	0.76	9.59 × 10^−3^	-	-	-	-
PC-O(34:3)	0.44	4.87 × 10^−7^	-	-	-	-	-	-
PC-O(36:3)	0.52	4.92 × 10^−6^	0.75	1.76 × 10^−3^	-	-	-	-
PC-O(36:5)	0.60	1.89 × 10^−4^	-	-	-	-	-	-
SM(32:1)	0.58	1.08 × 10^−4^	-	-	0.66	9.70 × 10^−3^	-	-
SM(39:1)	0.55	1.14 × 10^−4^	-	-	-	-	-	-
SM(41:2)	0.64	1.65 × 10^−4^	-	-	-	-	-	-

## Supplementary Materials

The following are available online at https://www.mdpi.com/2072-6694/12/11/3447/s1, Figure S1: The 15 enriched networks (A−O) identified by IPA in ovarian cancer patients vs. healthy controls based on metabolites and differential abundant proteins., Table S1: The metabolite result of Absolute IDQ p400 in 20 clinical samples., Table S2: Statistical analysis of metabolites between OC and HC., Table S3: The result of normalized protein abundance in the 20 samples., Table S4: Statistical analysis of proteins between OC and HC., Table S5: Enriched canonical pathway list in IPA knowledge base. Table S6: The 15 networks identified by IPA in ovarian cancer patients versus healthy controls.

## Abbreviations

CA-125	Cancer antigen 125
FIA	Flow injection analysis
OC	Ovarian cancer
HC	Healthy control
LOD	Limit of determination
AC	Acylcarnitines
DC	Diglycerides
LPS	Lysophosphatidylcholines
PC	Phosphatidylcholines
SM	Sphingomyelins
DAM	Differential abundant plasma metabolite
ROC	Receiver operating characteristic
AUC	Area under the curve
LFQ	Label free quantification
PCA	Principle component analysis
DAP	Differential abundant plasma protein
IGF	Insulin-like growth factor
IPA	Ingenuity pathway analysis
HIF-1	Hypoxia-inducible factor 1
DIABLO	Data Integration Analysis and Biomarker discovery using Latent cOmponents
KM	Kaplan–Meier
OS	Overall survival
DFS	Disease-free survival
PPCS	Phosphopantothenate–cysteine ligase
PMP2	Myelin P2 protein
TUBB	Tubulin beta chain
AC.0.0	L-carnitine
HR	Hazard ratio
ESI-LC–MS/MS	Electrospray ionization liquid chromatography–tandem mass spectrometry
FIA–MS/MS	Flow injection analysis–tandem mass spectrometry
MARS14	Multiple Affinity Removal Column Human 14
m/z	Mass to charge ratio

## References

[1] National Cancer Institute SEER Cancer Statistics Review 1975–2015. https://seer.cancer.gov/csr/1975_2015/results_merged/topic_survival.pdf.

[2] Force U.P.S.T., Grossman D.C., Curry S.J., Owens D.K., Barry M.J., Davidson K.W., Doubeni C.A., Epling J.W., Kemper A.R., Krist A.H. (2018). Screening for Ovarian Cancer. JAMA.

[3] Drescher C.W., Anderson G.L. (2018). The Yet Unrealized Promise of Ovarian Cancer Screening. JAMA Oncol..

[4] Menon U., Karpinskyj C., Gentry-Maharaj A. (2018). Ovarian Cancer Prevention and Screening. Obstet. Gynecol..

[5] Heiden M.G.V., DeBerardinis R.J. (2017). Understanding the Intersections between Metabolism and Cancer Biology. Cell.

[6] Cairns R.A., Harris I.S., Mak T.W. (2011). Regulation of cancer cell metabolism. Nat. Rev. Cancer.

[7] Li H., Ning S., Ghandi M., Kryukov G.V., Gopal S., Deik A., Souza A., Pierce K., Keskula P., Hernandez D. (2019). The landscape of cancer cell line metabolism. Nat. Med..

[8] Garg G., Yilmaz A., Kumar P., Turkoglu O., Mutch D.G., Powell M.A., Rosen B., Bahado-Singh R.O., Graham S.F. (2018). Targeted metabolomic profiling of low and high grade serous epithelial ovarian cancer tissues: A pilot study. Metabolomics.

[9] Li J., Xie H., Li K., Cheng J., Yang K., Wang J., Wang W., Zhang F., Li Z., Dhillon H.S. (2016). Distinct plasma lipids profiles of recurrent ovarian cancer by liquid chromatography-mass spectrometry. Oncotarget.

[10] Buas M.F., Gu H., Djukovic D., Zhu J., Drescher C.W., Urban N., Raftery D., Li C.I. (2015). Identification of novel candidate plasma metabolite biomarkers for distinguishing serous ovarian carcinoma and benign serous ovarian tumors. Gynecol. Oncol..

[11] Plewa S., Horała A., Dereziński P., Nowak-Markwitz E., Matysiak J., Kokot Z.J. (2019). Wide spectrum targeted metabolomics identifies potential ovarian cancer biomarkers. Life Sci..

[12] Bachmayr-Heyda A., Aust S., Auer K., Meier S.M., Schmetterer K.G., Dekan S., Gerner C., Pils D. (2016). Integrative Systemic and Local Metabolomics with Impact on Survival in High-Grade Serous Ovarian Cancer. Clin. Cancer Res..

[13] Zhang B., Barekati Z., Kohler C., Radpour R., Asadollahi R., Holzgreve W., Zhong X.Y. (2010). Proteomics and biomarkers for ovarian cancer diagnosis. Ann. Clin. Lab. Sci..

[14] Enroth S., Berggrund M., Lycke M., Broberg J., Lundberg M., Assarsson E., Olovsson M., Stålberg K., Sundfeldt K., Gyllensten U. (2019). High throughput proteomics identifies a high-accuracy 11 plasma protein biomarker signature for ovarian cancer. Commun. Biol..

[15] Dufresne J., Bowden P., Thavarajah T., Florentinus-Mefailoski A., Chen Z.Z., Tucholska M., Norzin T., Ho M.T., Phan M., Mohamed N. (2018). The plasma peptides of ovarian cancer. Clin. Proteom..

[16] Cheng Y., Liu C., Zhang N., Wang S., Zhang Z. (2014). Proteomics Analysis for Finding Serum Markers of Ovarian Cancer. BioMed Res. Int..

[17] Song G., Chen L., Zhang B., Song Q., Yu Y., Moore C., Wang T.-L., Shih I.-M., Zhang H., Chan D.W. (2018). Proteome-wide Tyrosine Phosphorylation Analysis Reveals Dysregulated Signaling Pathways in Ovarian Tumors. Mol. Cell. Proteom..

[18] Rai A.J., Zhang Z., Rosenzweig J., Shih I.-M., Pham T., Fung E.T., Sokoll L.J., Chan D.W. (2002). Proteomic approaches to tumor marker discovery. Arch. Pathol. Lab. Med..

[19] Hughes C.S., McConechy M.K., Cochrane D.R., Nazeran T., Karnezis A.N., Huntsman D.G., Morin G.B. (2016). Quantitative Profiling of Single Formalin Fixed Tumour Sections: Proteomics for translational research. Sci. Rep..

[20] Dieters-Castator D.Z., Rambau P.F., Kelemen L.E., Siegers G.M., Lajoie G.A., Postovit L.M., Köbel M. (2019). Proteomics-Derived Biomarker Panel Improves Diagnostic Precision to Classify Endometrioid and High-grade Serous Ovarian Carcinoma. Clin. Cancer Res..

[21] Song X., Ji J., Gleason K.J., Yang F., Martignetti J.A., Chen L.S., Wang P. (2019). Insights into Impact of DNA Copy Number Alteration and Methylation on the Proteogenomic Landscape of Human Ovarian Cancer via a Multi-omics Integrative Analysis. Mol. Cell. Proteom..

[22] The Cancer Genome Atlas Research Network (2011). Integrated genomic analyses of ovarian carcinoma. Nature.

[23] Zhang H., Liu T., Zhang Z., Payne S.H., Zhang B., McDermott J.E., Zhou J.-Y., Petyuk V.A., Chen L., Ray D. (2016). Integrated Proteogenomic Characterization of Human High-Grade Serous Ovarian Cancer. Cell.

[24] Ma W., Chen L.S., Özbek U., Han S.W., Lin C., Paulovich A.G., Zhong H., Wang P. (2019). Integrative Proteo-genomic Analysis to Construct CNA-protein Regulatory Map in Breast and Ovarian Tumors. Mol. Cell. Proteom..

[25] Worzfeld T., Finkernagel F., Reinartz S., Konzer A., Adhikary T., Nist A., Stiewe T., Wagner U., Looso M., Graumann J. (2018). Proteotranscriptomics Reveal Signaling Networks in the Ovarian Cancer Microenvironment. Mol. Cell. Proteom..

[26] Chong J., Soufan O., Li C., Caraus I., Li S., Bourque G., Wishart D.S., Xia J. (2018). MetaboAnalyst 4.0: Towards more transparent and integrative metabolomics analysis. Nucleic Acids Res..

[27] Reimand J., Arak T., Adler P., Kolberg L., Reisberg S., Peterson H., Vilo J. (2016). g:Profiler—a web server for functional interpretation of gene lists (2016 update). Nucleic Acids Res..

[28] Hu Q., Hisamatsu T., Haemmerle M., Cho M.S., Pradeep S., Rupaimoole R., Rodriguez-Aguayo C., Lopez-Berestein G., Wong S.T., Sood A.K. (2017). Role of Platelet-Derived Tgfβ1 in the Progression of Ovarian Cancer. Clin. Cancer Res..

[29] Holmes C.E., Levis J.E., Ornstein D.L. (2009). Activated platelets enhance ovarian cancer cell invasion in a cellular model of metastasis. Clin. Exp. Metastasis.

[30] Egan K., Crowley D., Smyth P., O’Toole S., Spillane C., Martin C., Gallagher M.F., Canney A., Norris L.A., Conlon N. (2011). Platelet Adhesion and Degranulation Induce Pro-Survival and Pro-Angiogenic Signalling in Ovarian Cancer Cells. PLoS ONE.

[31] Davis A.N., Afshar-Kharghan V., Sood A.K. (2014). Platelet Effects on Ovarian Cancer. Semin. Oncol..

[32] Cho M.S., Noh K., Haemmerle M., Celia M.S.L., Park H., Hu Q., Hisamatsu T., Mitamura T., Mak S.L.C., Kunapuli S. (2017). Role of ADP receptors on platelets in the growth of ovarian cancer. Blood.

[33] Wittamer V., Bondue B., Guillabert A., Vassart G., Parmentier M., Communi D. (2005). Neutrophil-Mediated Maturation of Chemerin: A Link between Innate and Adaptive Immunity. J. Immunol..

[34] Singel K.L., Emmons T.R., Khan A.N.H., Mayor P.C., Shen S., Wong J.T., Morrell K., Eng K.H., Mark J., Bankert R.B. (2019). Mature neutrophils suppress T cell immunity in ovarian cancer microenvironment. JCI Insight.

[35] Xintaropoulou C., Ward C., Wise A., Queckborner S., Turnbull A., Michie C.O., Williams A.R.W., Rye T., Gourley C., Langdon S.P. (2018). Expression of glycolytic enzymes in ovarian cancers and evaluation of the glycolytic pathway as a strategy for ovarian cancer treatment. BMC Cancer.

[36] Wang Z., Dong C. (2019). Gluconeogenesis in Cancer: Function and Regulation of PEPCK, FBPase, and G6Pase. Trends Cancer.

[37] Grasmann G., Smolle E., Olschewski H., Leithner A. (2019). Gluconeogenesis in cancer cells – Repurposing of a starvation-induced metabolic pathway?. Biochim. Biophys. Acta (BBA) Bioenerg..

[38] Wang X., Wang E., Kavanagh J.J., Freedman R.S. (2005). Ovarian cancer, the coagulation pathway, and inflammation. J. Transl. Med..

[39] Koh S.C., Tham K.-F., Razvi K., Oei P.-L., Lim F.-K., Roy A.-C., Prasad R. (2001). Hemostatic and Fibrinolytic Status in Patients With Ovarian Cancer and Benign Ovarian Cysts: Could D-dimer and Antithrombin III Levels Be Included as Prognostic Markers for Survival Outcome?. Clin. Appl. Thromb..

[40] Yu G., Qu G. (2017). High molecular weight caldesmon expression in ovarian adult granulosa cell tumour and fibrothecoma. Histopathology.

[41] McKenzie A.J., Hicks S.R., Svec K.V., Naughton H., Edmunds Z.L., Howe A.K. (2018). The mechanical microenvironment regulates ovarian cancer cell morphology, migration, and spheroid disaggregation. Sci. Rep..

[42] Dai C., Dai S., Cao J. (2012). Proteotoxic stress of cancer: Implication of the heat-shock response in oncogenesis. J. Cell. Physiol..

[43] Echo A., Howell V.M., Colvin E.K. (2015). The Extracellular Matrix in Epithelial Ovarian Cancer – A Piece of a Puzzle. Front. Oncol..

[44] Yang X., Fraser M., Abedini M.R., Bai T., Tsang B.K. (2008). Regulation of apoptosis-inducing factor-mediated, cisplatin-induced apoptosis by Akt. Br. J. Cancer.

[45] Yang W., Shin H.-Y., Cho H., Chung J.-Y., Lee E.-J., Kim J.-H., Kang E.-S. (2020). TOM40 Inhibits Ovarian Cancer Cell Growth by Modulating Mitochondrial Function Including Intracellular ATP and ROS Levels. Cancers.

[46] Wang D., He J., Dong J., Meyer T.F., Xu T. (2020). The HIPPO pathway in gynecological malignancies. Am. J. Cancer Res..

[47] Hall C.A., Wang R., Miao J., Oliva E., Shen X., Wheeler T., Hilsenbeck S.G., Orsulic S., Goode S. (2010). Hippo Pathway Effector Yap Is an Ovarian Cancer Oncogene. Cancer Res..

[48] Zubor P., Dankova Z., Kolkova Z., Holubekova V., Brany D., Mersakova S., Samec M., Liskova A., Koklesova L., Kubatka P. (2020). Rho GTPases in Gynecologic Cancers: In-Depth Analysis toward the Paradigm Change from Reactive to Predictive, Preventive, and Personalized Medical Approach Benefiting the Patient and Healthcare. Cancers.

[49] Jeong K.J., Park S.Y., Cho K.H., Sohn J.S., Lee J., Kim Y.K., Kang J., Park C.G., Han J.W., Lee H.Y. (2019). Correction: The Rho/ROCK pathway for lysophosphatidic acid-induced proteolytic enzyme expression and ovarian cancer cell invasion. Oncogene.

[50] Hudson L.G., Gillette J.M., Kang H., Rivera M.R., Wandinger-Ness A. (2018). Ovarian Tumor Microenvironment Signaling: Convergence on the Rac1 GTPase. Cancers.

[51] Ose J., Fortner R.T., Schock H., Peeters P.H., Onland-Moret N.C., Bueno-De-Mesquita H.B., Weiderpass E., Gram I.T., Overvad K., Tjonneland A. (2014). Insulin-like growth factor I and risk of epithelial invasive ovarian cancer by tumour characteristics: Results from the EPIC cohort. Br. J. Cancer.

[52] Amutha P., Rajkumar T. (2017). Role of Insulin-like Growth Factor, Insulin-like Growth Factor Receptors, and Insulin-like Growth Factor-binding Proteins in Ovarian Cancer. Indian J. Med. Paediatr. Oncol..

[53] Wang J., Zhou J.-Y., Wu G.S. (2007). ERK-Dependent MKP-1 Mediated Cisplatin Resistance in Human Ovarian Cancer Cells. Cancer Res..

[54] Maihle N.J., Baron A.T., Barrette B.A., Boardman C.H., Christensen T.A., Cora E.M., Faupel-Badger J.M., Greenwood T., Juneja S.C., Lafky J.M. (2002). EGF/ErbB Receptor Family in Ovarian Cancer. Infect. Complicat. Cancer Patients.

[55] Sheng Q., Liu J. (2011). The therapeutic potential of targeting the EGFR family in epithelial ovarian cancer. Br. J. Cancer.

[56] Hayden M.S., Ghosh S. (2008). Shared Principles in NF-κB Signaling. Cell.

[57] Salazar L., Kashiwada T., Krejci P., Meyer A.N., Casale M., Hallowell M., Wilcox W.R., Donoghue D.J., Thompson L.M. (2014). Fibroblast Growth Factor Receptor 3 Interacts with and Activates TGFβ-Activated Kinase 1 Tyrosine Phosphorylation and NFκB Signaling in Multiple Myeloma and Bladder Cancer. PLoS ONE.

[58] Sau A., Lau R., Cabrita M.A., Nolan E., Crooks P.A., Visvader J.E., Pratt M.C. (2016). Persistent Activation of NF-κB in BRCA1-Deficient Mammary Progenitors Drives Aberrant Proliferation and Accumulation of DNA Damage. Cell Stem Cell.

[59] Birner P., Schindl M., Obermair A., Breitenecker G., Oberhuber G. (2001). Expression of hypoxia-inducible factor 1alpha in epithelial ovarian tumors: Its impact on prognosis and on response to chemotherapy. Clin. Cancer Res..

[60] Muz B., De La Puente P., Azab F., Azab A.K. (2015). The role of hypoxia in cancer progression, angiogenesis, metastasis, and resistance to therapy. Hypoxia.

[61] Ween M.P., Oehler M.K., Ricciardelli C. (2012). Transforming Growth Factor-Beta-Induced Protein (TGFBI)/(βig-H3): A Matrix Protein with Dual Functions in Ovarian Cancer. Int. J. Mol. Sci..

[62] Yoshikawa T., Miyamoto M., Aoyama T., Soyama H., Goto T., Hirata J., Suzuki A., Nagaoka I., Tsuda H., Furuya K. (2018). JAK2/STAT3 pathway as a therapeutic target in ovarian cancers. Oncol. Lett..

[63] Wen W., Liang W., Wu J., Kowolik C.M., Buettner R., Scuto A., Hsieh M.-Y., Hong H., Brown C.E., Forman S.J. (2014). Targeting JAK1/STAT3 Signaling Suppresses Tumor Progression and Metastasis in a Peritoneal Model of Human Ovarian Cancer. Mol. Cancer Ther..

[64] Singh A., Shannon C.P., Gautier B., Rohart F., Vacher M., Tebbutt S.J., Cao K.-A.L. (2019). DIABLO: An integrative approach for identifying key molecular drivers from multi-omics assays. Bioinformatics.

[65] E Geyer P., Holdt L.M., Teupser D., Mann M. (2017). Revisiting biomarker discovery by plasma proteomics. Mol. Syst. Biol..

[66] Chen F., Shen J., Wang J., Cai P., Huang Y. (2018). Clinical analysis of four serum tumor markers in 458 patients with ovarian tumors: Diagnostic value of the combined use of HE4, CA125, CA19-9, and CEA in ovarian tumors. Cancer Manag. Res..

[67] Russell M.R., Graham C., D’Amato A., Gentry-Maharaj A., Ryan A., Kalsi J.K., Whetton A.D., Menon U., Jacobs I., Graham R.L.J. (2019). Diagnosis of epithelial ovarian cancer using a combined protein biomarker panel. Br. J. Cancer.

[68] Jiang W., Huang R., Duan C., Fu L., Xi Y., Yang Y., Yang W.-M., Yang N., Yang N.-H., Huang R.-P. (2013). Identification of Five Serum Protein Markers for Detection of Ovarian Cancer by Antibody Arrays. PLoS ONE.

[69] Visintin I., Feng Z., Longton G., Ward D.C., Alvero A.B., Lai Y., Tenthorey J., Leiser A., Flores-Saaib R., Yu H. (2008). Diagnostic Markers for Early Detection of Ovarian Cancer. Clin. Cancer Res..

[70] Mor G., Visintin I., Lai Y., Zhao H., Schwartz P., Rutherford T., Yue L., Bray-Ward P., Ward D.C. (2005). Serum protein markers for early detection of ovarian cancer. Proc. Natl. Acad. Sci. USA.

[71] Melone M.A.B., Valentino A., Margarucci S., Galderisi U., Giordano A., Peluso G. (2018). The carnitine system and cancer metabolic plasticity. Cell Death Dis..

[72] Fukushima N., Yokouchi K., Kuroiwa M., Kawagishi K., Moriizumi T. (2017). Acetyl- l -carnitine enhances myelination of regenerated fibers of the lateral olfactory tract. Neurosci. Lett..

[73] Fekete J.T., Ősz Á., Pete I., Nagy G.R., Vereczkey I., Győrffy B. (2020). Predictive biomarkers of platinum and taxane resistance using the transcriptomic data of 1816 ovarian cancer patients. Gynecol. Oncol..

[74] Wang L., Liu Y., Qi C., Shen L., Wang J., Liu X., Zhang N., Bing T., Shangguan D. (2018). Oxidative degradation of polyamines by serum supplement causes cytotoxicity on cultured cells. Sci. Rep..

[75] Voorde J.V., Ackermann T., Pfetzer N., Sumpton D., Mackay G., Kalna G., Nixon C., Blyth K., Gottlieb E., Tardito S. (2019). Improving the metabolic fidelity of cancer models with a physiological cell culture medium. Sci. Adv..

[76] Cuperlovic-Culf M., Barnett D.A., Culf A.S., Chute I. (2010). Cell culture metabolomics: Applications and future directions. Drug Discov. Today.

[77] Sørensen B.H., Thorsteinsdottir U.A., Lambert I.H. (2014). Acquired cisplatin resistance in human ovarian A2780 cancer cells correlates with shift in taurine homeostasis and ability to volume regulate. Am. J. Physiol. Physiol..

[78] Sperner-Unterweger B., Neurauter G., Klieber M., Kurz K., Meraner V., Zeimet A., Fuchs D. (2011). Enhanced tryptophan degradation in patients with ovarian carcinoma correlates with several serum soluble immune activation markers. Immunobiology.

[79] Lanser L., Kink P., Egger E.M., Willenbacher W., Fuchs D., Weiss G., Kurz K. (2020). Inflammation-Induced Tryptophan Breakdown is Related With Anemia, Fatigue, and Depression in Cancer. Front. Immunol..

[80] Ye C., Geng Z., Dominguez D., Chen S., Fan J., Qin L., Long A., Zhang Y., Kuzel T.M., Zhang B. (2016). Targeting Ornithine Decarboxylase by α-Difluoromethylornithine Inhibits Tumor Growth by Impairing Myeloid-Derived Suppressor Cells. J. Immunol..

[81] Kim H.I., Schultz C.R., Buras A., Friedman E., Fedorko A.M., Seamon L., Chandramouli G.V.R., Maxwell G.L., Bachmann A.S., Risinger J.I. (2017). Ornithine decarboxylase as a therapeutic target for endometrial cancer. PLoS ONE.

[82] Chen Y., Zhuang H., Chen X., Shi Z., Wang X. (2018). Spermidine-induced growth inhibition and apoptosis via autophagic activation in cervical cancer. Oncol. Rep..

[83] Madeo F., Eisenberg T., Pietrocola F., Kroemer G. (2018). Spermidine in health and disease. Science.

[84] A Zeleznik O., Clish C.B., Kraft P., Avila-Pancheco J., Eliassen A.H., Tworoger S.S. (2020). Circulating Lysophosphatidylcholines, Phosphatidylcholines, Ceramides, and Sphingomyelins and Ovarian Cancer Risk: A 23-Year Prospective Study. J. Natl. Cancer Inst..

[85] Law S.-H., Chan M.-L., Marathe G.K., Parveen F., Chen C.-H., Ke L.-Y. (2019). An Updated Review of Lysophosphatidylcholine Metabolism in Human Diseases. Int. J. Mol. Sci..

[86] Curtarello M., Tognon M., Venturoli C., Silic-Benussi M., Grassi A., Verza M., Minuzzo S., Pinazza M., Brillo V., Tosi G. (2019). Rewiring of Lipid Metabolism and Storage in Ovarian Cancer Cells after Anti-VEGF Therapy. Cells.

[87] Ridgway N.D. (2013). The role of phosphatidylcholine and choline metabolites to cell proliferation and survival. Crit. Rev. Biochem. Mol. Biol..

[88] Iorio E., Ricci A., Bagnoli M., Pisanu M.E., Castellano G., Di Vito M., Venturini E., Glunde K., Bhujwalla Z.M., Mezzanzanica D. (2010). Activation of Phosphatidylcholine Cycle Enzymes in Human Epithelial Ovarian Cancer Cells. Cancer Res..

[89] Spadaro F., Ramoni C., Mezzanzanica D., Miotti S., Alberti P., Cecchetti S., Iorio E., Dolo V., Canevari S., Podo F. (2008). Phosphatidylcholine-Specific Phospholipase C Activation in Epithelial Ovarian Cancer Cells. Cancer Res..

[90] Podo F., Paris L., Cecchetti S., Spadaro F., Abalsamo L., Ramoni C., Ricci A., Pisanu M.E., Sardanelli F., Canese R. (2016). Activation of Phosphatidylcholine-Specific Phospholipase C in Breast and Ovarian Cancer: Impact on MRS-Detected Choline Metabolic Profile and Perspectives for Targeted Therapy. Front. Oncol..

[91] Slotte J.P. (2013). Biological functions of sphingomyelins. Prog. Lipid Res..

[92] Kreitzburg K.M., Van Waardenburg R.C.A.M., Yoon K.J. (2018). Sphingolipid metabolism and drug resistance in ovarian cancer. Cancer Drug Resist..

[93] Huang H., Tong T.-T., Yau L.-F., Chen C.-Y., Mi J.-N., Wang J.-R., Jiang Z.-H. (2016). LC-MS Based Sphingolipidomic Study on A2780 Human Ovarian Cancer Cell Line and its Taxol-resistant Strain. Sci. Rep..

[94] Nunes S.C., Serpa J. (2018). Glutathione in Ovarian Cancer: A Double-Edged Sword. Int. J. Mol. Sci..

[95] Pellerin D., Gagnon H., Dubé J., Corbin F. (2015). Amicon-adapted enhanced FASP: An in-solution digestion-based alternative sample preparation method to FASP. F1000Research.

[96] Perez-Riverol Y., Csordas A., Bai J., Bernal-Llinares M., Hewapathirana S., Kundu D.J., Inuganti A., Griss J., Mayer G., Eisenacher M. (2019). The PRIDE database and related tools and resources in 2019: Improving support for quantification data. Nucleic Acids Res..

[97] (2019). The UniProt Consortium UniProt: A worldwide hub of protein knowledge. Nucleic Acids Res..

[98] Tyanova S., Cox J. (2018). Perseus: A Bioinformatics Platform for Integrative Analysis of Proteomics Data in Cancer Research. Methods Mol. Biol..

